# 2501: Depression and mental health service use: Data from National Health and Nutrition Examination Survey (NHANES) 2006–2012

**DOI:** 10.1017/cts.2017.287

**Published:** 2018-05-10

**Authors:** Magda Shaheen, Senait Teklehaimanot

**Affiliations:** David Geffen School of Medicine, UCLA , Los Angeles, CA, USA

## Abstract

OBJECTIVES/SPECIFIC AIMS: Examine mental health service use and its correlates among depressed group in a national sample of population≥20 years old. METHODS/STUDY POPULATION: Analysis of data for adult≥20 years old from the NHANES 2006–2012. Depression was assessed using the 9-item PHQ. The use of mental health and antidepressant drug were used to indicate the service use. We utilized multiple logistic regressions to determine the independent association between service use and each independent variable (demographics, health status, food security, chronic conditions, and depression severity) controlling for other independent variables. Data were presented as adjusted odds ratio (AOR), 95% confidence interval (95% CI), and *p*-value of statistical significance. *p*-value of<0.05 indicates statistical significance. RESULTS/ANTICIPATED RESULTS: Of the 17,824 subjects, 22% had mild to severe depression. Among the depressed group, 25% used antidepressant, 17% used mental health service. For the use of mental health services among the depressed group, African-American (AA), ≥60 years old, uninsured and foreign born were less likely to use the mental health service relative to other groups [AOR=0.58 (95% CI=0.45–0.75), 0.21 (95% CI=0.14–0.33), 0.61 (95% CI=0.45–0.83), 0.41 (95% CI=0.17–0.99), respectively, *p*<0.05]. For the use of antidepressant drug among the depressed group, AA, Hispanics, uninsured and foreign born were less likely to use antidepressant drug relative to other groups [OR=0.26 (95% CI=0.20–0.33), 0.42 (95% CI=0.31–0.57), 0.41 (95% CI=0.31–0.56), 0.20 (95% CI=0.10–0.78), respectively, *p*<0.05). For the use of mental health services and/or antidepressant drug among the depressed group, 40–59 years old, AA, Hispanics, uninsured, foreign born were less likely to use mental health services and/or antidepressant drug relative to other groups [OR=0.52 (95% CI=0.38–0.72), 0.35 (95% CI=0.28–0.43), 0.52 (95% CI=0.40–0.69), 0.53 (95% CI=0.41–0.68), 0.30 (95% CI=0.13–0.68), respectively, *p*<0.05). DISCUSSION/SIGNIFICANCE OF IMPACT: Our study showed that minority (AA and Hispanics), foreign born and uninsured with depression were less likely to use mental health services and/or antidepressant drug relative to other groups. Culturally and linguistically adapted intervention that involves community and providers to increase awareness about depression and the available services/treatment among minority, immigrant, and uninsured population are needed.

